# Depletion of exosomal circLDLR in follicle fluid derepresses miR-1294 function and inhibits estradiol production via CYP19A1 in polycystic ovary syndrome

**DOI:** 10.18632/aging.103602

**Published:** 2020-07-10

**Authors:** Xin Huang, Bi Wu, Miaoxin Chen, Ling Hong, Pengcheng Kong, Zhiyun Wei, Xiaoming Teng

**Affiliations:** 1 Department of Assisted Reproduction, and Clinical and Translational Research Center, Shanghai First Maternity and Infant Hospital, Tongji University School of Medicine, Shanghai, China

**Keywords:** PCOS, exosome, folliculogenesis, circLDLR, CYP19A1

## Abstract

Polycystic ovary syndrome (PCOS) is a common endocrine and metabolic disorder in reproductive women and is characterized by polycystic ovaries, hyperandrogenism and chronic anovulation. Abnormal folliculogenesis is considered as a common characteristic of PCOS. Our aim is to identify the altered circRNA expression profile in exosomes isolated from follicular fluid (FF) of PCOS patients to investigate the molecular function of exosomal circRNA, as a vital mediator in follicular microenvironment, in the aetiology and pathobiology of PCOS. In this study, the circRNA expression profile of FF exosomes were compared between PCOS and control patients by RNA sequencing (N=5 vs 5). Sixteen circRNAs showed significantly different expression. GO and KEGG pathway analyses indicated that their parental genes were enriched in PCOS-related pathways, including ovarian steroidogenesis, aldosterone synthesis and secretion, and Jak-STAT signaling. Among sixteen differentially expressed circRNAs, hsa_circ_0006877 (circLDLR) was processed from its parental LDLR (low density lipoprotein receptor) transcript, which participated in ovarian steroidogenesis. Its depletion in PCOS FF exosomes was further verified in an additional cohort (N=25 vs 25) by qRT-PCR. And a circLDLR-miR-1294-CYP19A1 competing endogenous RNA (ceRNA) network was predicted by cytoscape software, and confirmed by luciferase assay and correlative expression in the cumulus cells of PCOS patients. Mechanistically, the intercellular transfer of functional circLDLR assay and its withdrawal experiments in KGN cells showed that depleting circLDLR in exosomes increased miR-1294 expression and inhibited CYP19A1 expression in recipient cells, as well as reduced their estrogen (E2) secretion. Our findings revealed a ceRNA network of circLDLR and provided new information on abnormal follicle development in PCOS.

## INTRODUCTION

Polycystic ovary syndrome (PCOS) is the most common and complex endocrinopathy. Due to PCOS, 5% to 10% of women in reproductive-age are infertile [1]. It is a multi-factorial and heterogeneous syndrome with variable phenotypes, including hyperandrogenism, menstrual irregularity and polycystic ovarian morphology [2]. The health risks associated with PCOS, however, go far beyond management of these common presenting symptoms or fertility treatment and likely extend past the reproductive years through and beyond menopause. With increasing ages, patients with PCOS often have an increased risk of developing metabolic disorders, such as type 2 diabetes (T2D), insulin resistance, obesity [3] and cardiovascular disease [4, 5].

Although the clinical and biochemical characteristics of PCOS have typical heterogeneity, abnormal follicular development to induce anovulation is still the basic important characteristic of PCOS [6, 7]. Follicular fluid (FF) provides an important microenvironment for follicular development and oocyte maturation. It is the medium for bi-directional communication between oocytes and the surrounding cells [8]. The major components of follicular fluid are proteins, steroids, metabolites and polysaccharides [9, 10], which are secreted by granulosa cells, theca cells and oocytes or diffused from capillaries [11]. Recently, an increasing number of studies have shown that exosomes are present in FF and act as message transmitters in intercellular communication by transferring a variety of proteins, lipids, miRNAs, and circRNAs [12–16].

Exosomes are small membrane-enclosed vesicles (30-200 nm in diameter) secreted by a wide range of living cells under normal or pathophysiological circumstances [17]. To date, there have been limited researches focusing on intercellular communication mediated by exosomes inside the ovarian follicle, which mainly underline the involvement of exosomal miRNAs in FF during several phases of the reproductive process. In 2012, da Silveira et al., for the first time, described the presence of exosomes containing miRNAs in equine FF [18] and further discussed the role of hormone-regulated miRNAs within different ovarian follicular cells [19]. After that, exosomal miRNAs from bovine FF were isolated and demonstrated to be able to support cumulus expansion [20, 21].

CircRNAs, as a new class of abundant and stable endogenous noncoding RNAs [22], have been proven to play diverse biological roles such as miRNA sponges in regulating gene expression [23, 24]. Unlike linear RNA, circRNA was characterized by covalently closed loop without 5’ or 3’ polarity [23]. The special covalently closed loop structure makes it more stable than traditional linear RNAs against RNase and exonuclease to exert its biological function for a long period of time [25]. With the development of microarray and high throughput sequencing and related bioinformatic analysis, more and more circRNAs have been identified and proven to involve in the progression of various cancers [26–29]. Notably, just recently, differentially expressed circRNAs have been revealed in PCOS tissues using microarray or RNA sequencing [15, 30–32]. Although these studies were lack of functional analysis or verification, their results paved the way for further research on the roles of circRNAs in the etiology and pathogenesis of PCOS. In this study, we established the circRNA expression profiles of exosomes isolated from follicular fluid of PCOS using RNA sequencing technology and explored the functions and mechanism of circLDLR in abnormal folliculogenesis of PCOS.

## RESULTS

### Isolation and identification of exosomes

**Figure 1:**
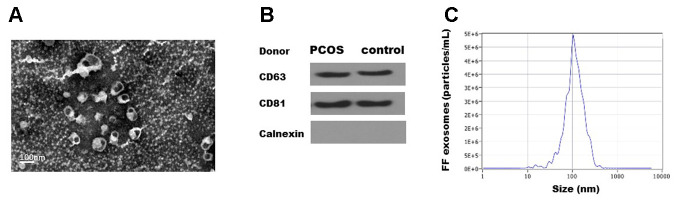
**Characterization of exosomes isolated from human follicular fluid.** (**A**). Transmission electron micrographs (TEM) of exosomes isolated from follicular fluid. Scale bar, 100 nm. (**B**) Immunoblots of positive exosomal markers (CD63, CD81) and negative marker (Calnexin) in representative follicular fluid exosomes. (**C**) The representative nanoparticle tracking analysis (NTA) profile of isolated exosomes.

Exosomes were isolated from the follicular fluid of both PCOS and control patients using ExoQuick. Transmission electron microscopy (TEM) showed that FF exosomes exhibited round-shaped morphologies and diameters ranging 40-100 nm (Figure 1A. Moreover, the presence of the exosome markers CD63, CD81 were confirmed by Western blot (Figure 1B). The size distribution measured by NTA also showed a typical profile of exosomes (Figure 1C). TEM, NTA and Western blot results were consistent with the characteristics of exosomes.

### Differential analysis of exosomal circRNAs between PCOS patients and control individuals

**Figure 2:**
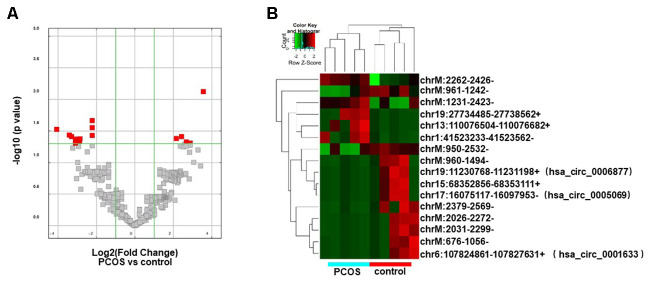
**Differentially expressed circRNA analysis.** (**A**) Volcano plot showing the distribution of circRNA differential expression according to their *p* values and fold-changes. Candidates with p< 0.05 and fold change >= 2 are highlighted in red. (**B**) Heat map showing hierarchical clustering analysis of circRNA candidates in follicular exosomes isolated from PCOS and control donors.

The circRNA expression profiles of exosomes isolated from FF in PCOS (N=5) and control patients (N=5) were examined by RNA sequencing. The raw sequencing data were deposited in NCBI’s Gene Expression Omnibus (GEO; http://www.ncbi.nlm.nih.gov/geo/) and can be accessed through the GEO series accession number (GSE145461). The circRNA expression profiles were comparatively analyzed in the two groups of exosomes (exo-PCOS and exo-control). A circRNA candidate was considered to be differentially expressed if its levels between both experiment groups showed a statistically significant difference (p<0.05) of at least two-fold. Based on this criterion, we identified 16 differentially expressed circRNAs (5 up-regulated and 11 down-regulated ones, Figure 2A) between exo-PCOS and exo-control, including 3 annotated exonic circRNAs and 13 novel ones (Table 1). Clustering analysis based on the differentially expressed circRNAs clustered the exo-PCOS and the exo-control groups (Figure 2B).

**Table 1:** The differentially expressed circRNAs in exosome derived from PCOS follicular fluid (p<0.05).

	**CircRNA ID**	**FC**	**circBaseID**	**best_transcript**	**Gene Name**	**Catalog**	**Predicted Sequence length**
UP	chrM:2262-2426-	11.85	novel	uc004cos.5	TVAS5	antisense	165
	chr13:110076504-110076682+	7.351	novel	/	/	intergenic	179
	chr19:27734485-27738562+	6.399	novel	/	/	intergenic	4078
	chrM:1231-2423-	5.372	novel	uc004cor.1	DQ582265	antisense	1193
	chr1:41523233-41523562-	4.472	novel	ENST00000326197	SCMH1	intronic	330
Down	chrM:961-1242-	16.79	novel	ENST00000389680	MT-RNR1	antisense	282
	chrM:2026-2272-	10.71	novel	uc004cos.5	TVAS5	antisense	247
	chr15:68352856-68353111+	8.513	novel	ENST00000249636	PIAS1	intronic	256
	chrM:676-1056-	8.513	novel	ENST00000389680	MT-RNR1	antisense	381
	chr17:16075117-16097953-	8.037	hsa_circ_0005069	NM_006311	NCOR1	exonic	505
	chr19:11230768-11231198+	7.411	hsa_circ_0006877	NM_000527	LDLR	exonic	295
	chr6:107824861-107827631+	7.256	hsa_circ_0001633	NM_018013	SOBP	exonic	325
	chrM:950-2532-	4.676	novel	uc004cor.1	DQ582265	antisense	1583
	chrM:960-1494-	4.676	novel	ENST00000389680	MT-RNR1	antisense	535
	chrM:2379-2569-	4.676	novel	uc004cos.5	TVAS5	antisense	191
	chrM:2031-2299-	9.803	novel	uc004cos.5	TVAS5	antisense	269

FC: fold change.

### Functional analysis of the differentially expressed circRNAs

**Figure 3:**
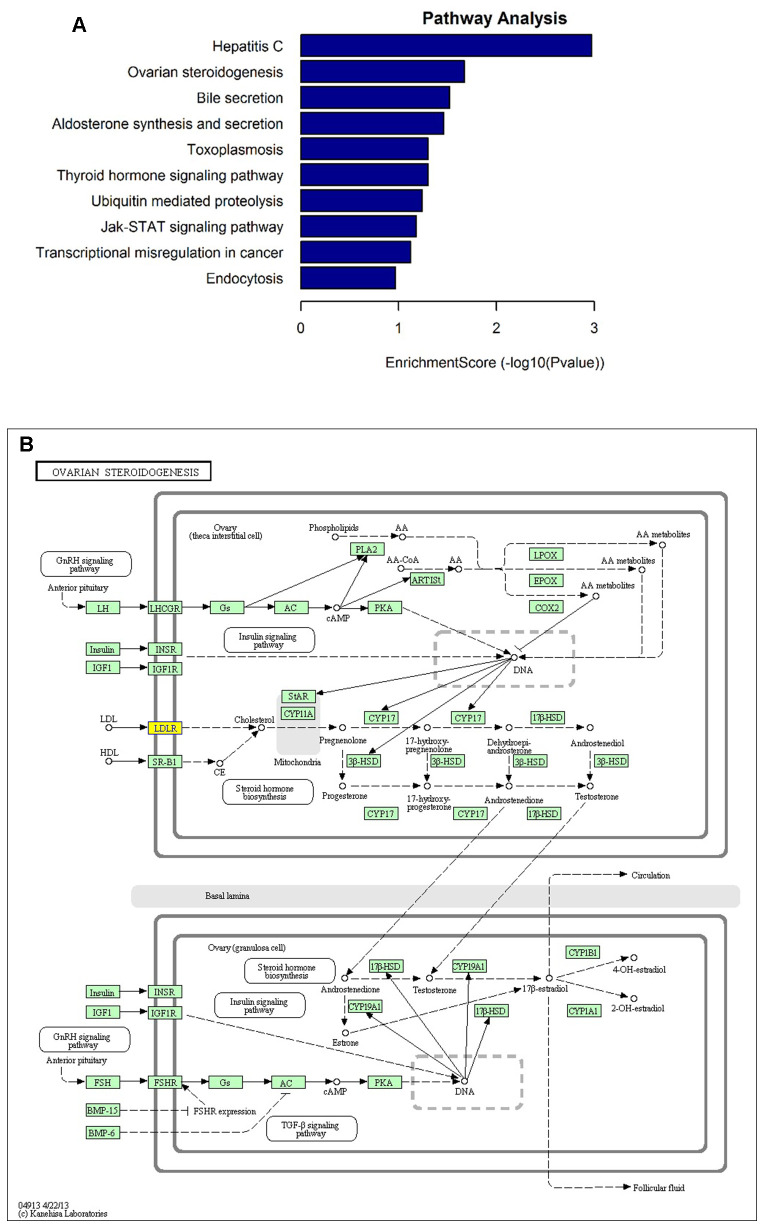
**KEGG pathway analysis of the parental genes of differentially expressed circRNA candidates.** (**A**) The most significantly enriched pathways: ovarian steroidogenesis, aldosterone synthesis and secretion, thyroid hormone signaling pathway, ubiquitin mediated proteolysis, Jak-STAT signaling pathway, and endocytosis. (**B**) The diagrammatic sketch of ovarian steroidogenesis pathway in which circLDLR (hsa_circ_0006877) is involved.

To interpret the pathological relevance of the differentially expressed circRNAs, GO and KEGG pathway analysis were performed on their parental genes. Regarding GO annotation, the biological processes, molecular function and cellular components associated with the coding genes of dysregulated circRNAs are presented in Supplementary Tables 2 and 3, respectively. The GO analysis showed that the most significantly altered biological processes included cholesterol homeostasis, sterol homeostasis, lipid homeostasis, regulation of cellular catabolic process, lipid transport. Moreover, the KEGG pathway analysis demonstrated that the most significantly altered pathways included ovarian steroidogenesis, aldosterone synthesis and secretion, thyroid hormone signaling pathway, ubiquitin mediated proteolysis, Jak-STAT signaling pathway, and endocytosis (Figure 3A and Supplementary Table 4). Interestingly, one of them, hsa_circ_0006877 (circLDLR), was processed from its parental LDLR (low density lipoprotein receptor) gene, which participated in ovarian steroidogenesis pathway (Figure 3B).

### Differential expression validation by qRT-PCR

**Figure 4:**
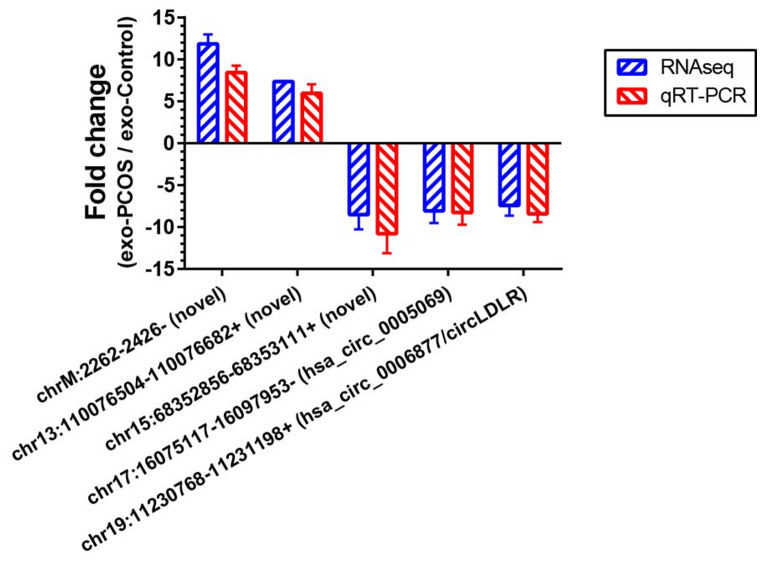
**Validation of the RNA sequencing results by qRT-PCR.** Five differentially expressed circRNAs (chr17:16075117-16097953-/ hsa_circ_0005069, chr19:11230768-11231198+/ hsa_circ_0006877, chr15:68352856-68353111+, chrM:2262-2426- and chr13:110076504-110076682+) were validated by qRT-PCR. The red bars show the relative gene expression measured using qRT-PCR. The blue bars show the relative gene expression measured using RNA sequencing. N=5 vs 5 for RNA sequencing, and N=25 vs 25 for qRT-PCR.

To further validate our circRNA profiling results, the exosomes isolated from 50 additional FF samples (25 PCOS and 25 controls), were used to extract the RNA for qRT-PCR. Five altered circRNAs (FC>7 based on sequencing data) were selected for validation, including two down-regulated annotated circRNAs (chr17:16075117-16097953-/ hsa_circ_0005069 and chr19:11230768-11231198+/ circLDLR), one down-regulated novel circRNA (chr15:68352856-68353111+), and two up-regulated novel circRNAs (chrM:2262-2426- and chr13:110076504-110076682+). The qRT-PCR results confirmed their presence in all of the samples, and validated their differential expression in exo-PCOS, consistently with the RNA sequencing data (Figure 4).

### Construction of a circLDLR-regulated ceRNA network

**Figure 5:**
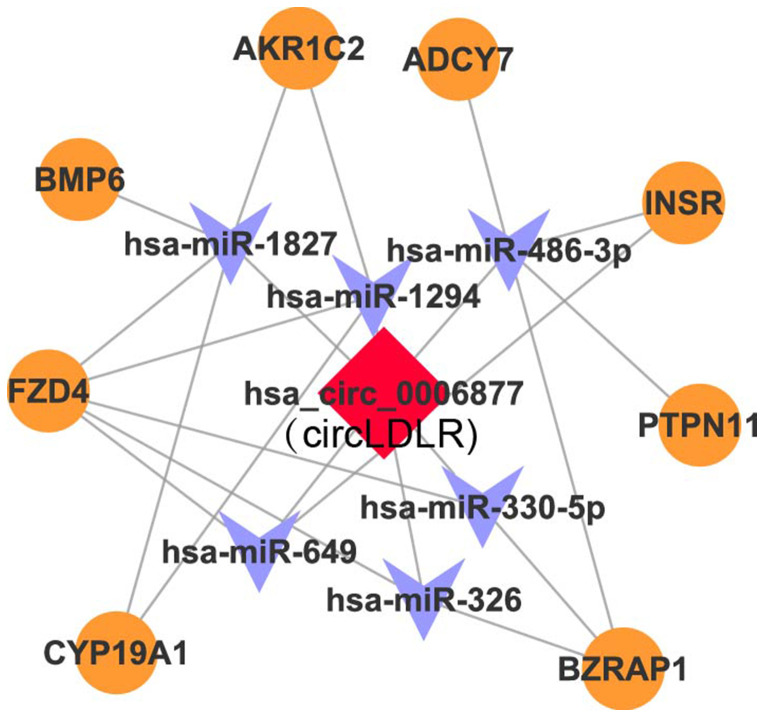
**The circLDLR-mediated ceRNA network.** The ceRNA network of circLDLR (has_circ_0006877) was computationally constructed to predict the most likely sponged miRNAs and their affected targets. In the network, (1) the most likely putative miRNAs (top 6) with MREs on hsa_circ_0006877 were selected, including has-miR-1294, hsa-miR-1827, hsa-miR-326, hsa-miR-330-5p, hsa-miR-486-3p, and hsa-miR-649; (2) the target genes of putative miRNAs, which were also involved in ovarian steroidogenesis pathway, were selected, including CYP19A1, BMP6, INSR, AKRIC2, ADCY7, FZD4, PTPN11, and BZRAP1. Hsa_circ_0006877 was indicated in red; miRNAs in blue; and mRNAs in orange.

Recent studies have demonstrated that circRNAs can absorb miRNAs by stable complementary binding and serve as miRNA sponges to regulate gene expression [33, 34]. Given that circRNAs can interact with miRNAs through their miRNA recognition elements (MREs) within a ceRNA network [35], we searched for putative miRNAs with MREs in circLDLR using miRanda software. A total of 162 miRNAs were predicted to possess MREs in circLDLR. And the top 6 putative binding miRNAs are has-miR-1294, hsa-miR-1827, hsa-miR-326, hsa-miR-330-5p, hsa-miR-486-3p, and hsa-miR-649 (Supplementary Table 5). Intersecting the potential target mRNAs of these top 6 putative miRNAs with the genes involved in ovarian steroidogenesis pathways, we found 8 mRNAs, i.e. CYP19A1, BMP6, INSR, AKRIC2, ADCY7, FZD4, PTPN11, BZRAP1. Thus, a credible circLDLR-miRNA-mRNA ceRNA network was constructed (Figure 5 and Supplementary Table 6). Within the ceRNA network, we found that the CYP19A1 might be an interesting target of miR-1294. CYP19A1, which encodes cytochrome P450 aromatase that converts androgens to 17-β estradiol (E2), is considered to be one of the essential genes in the follicular arrest in PCOS [36]. Several studies have proven lower expression level of CYP19A1, as well as reduction of ovarian aromatase activity and E2 production in PCOS [37–39]. Therefore, we deduce that the circLDLR-miR-1294-CYP19A1 ceRNA network is more likely to play an important role in regulating E2 production.

### Co-expression characteristics of circLDLR-miR-1294-CYP19A1 ceRNA network in PCOS granulosa cells

**Figure 6:**
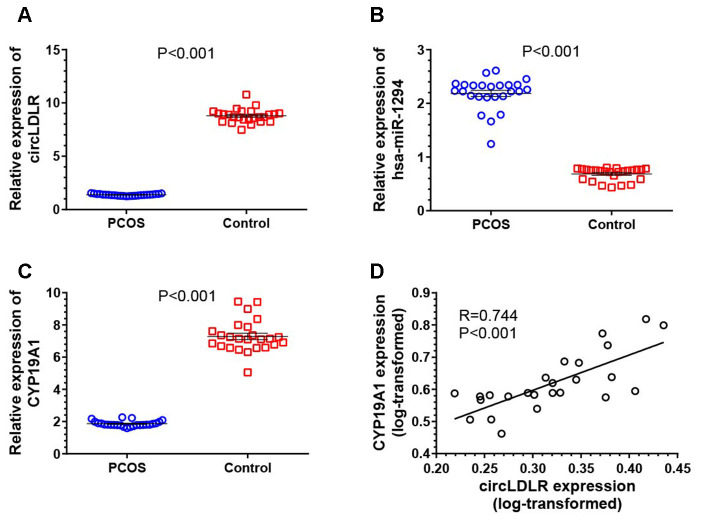
**Transcriptional dysregulation of circLDLR-miR-1294-CYP19A1 ceRNA network in granulosa cells of PCOS.** The abundance of circLDLR (**A**), miR-1294 (**B**) and CYP19A1 (**C**) were compared between PCOS and non-PCOS control by qRT-PCR. (**D**) Expression correlation between circLDLR and CYP19A1. GAPDH was used as the internal control for circLDLR and CYP19A1, while U6 was used as the internal control for miR-1294. N=25 vs 25. The results were presented as mean ± SEM.

The expression of the network partners (circLDLR, miR-1294 and CYP19A1) in granulosa cells of PCOS (N=25) and control (N=25) patients were quantified by qRT-PCR to validate the potential co-expression. Compared to controls, the expression levels of circLDLR and CYP19A1 were significantly decreased in PCOS samples, with 6.38- and 3.92- fold decrease observed, respectively (Figure 6A, 6C). The expression level of miR-1294 in PCOS samples was significantly higher (3.19-fold) than that in the control group (Figure 6B). A significant positive correlation was observed between circLDLR and CYP19A1 (Figure 6D). The co-expression characteristics observed were accordant with the ceRNA hypothesis.

### Confirmation of the direct interactions between the partners within the circLDLR-miR-1294-CYP19A1 ceRNA network

**Figure 7:**
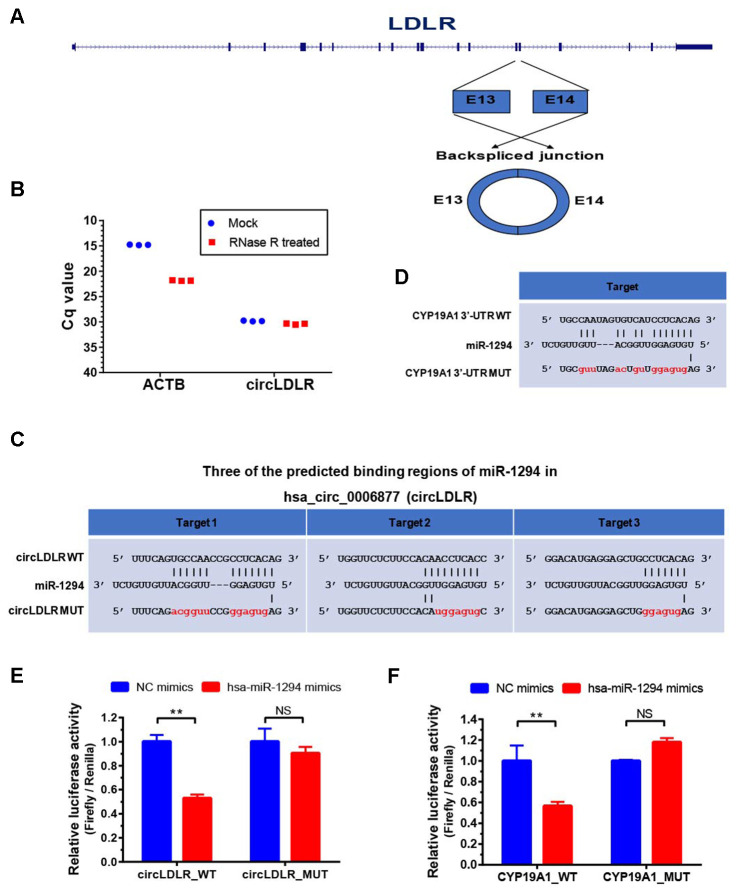
**Validation of direct targeting between the partners within the circLDLR-miR-1294-CYP19A1 ceRNA network.** (**A**) The sketch map of LDLR gene and its derived circLDLR (has_circ_0006877). The circLDLR is formed by backsplicing junction of the 13 and 14 exons of LDLR. (**B**) The RNase R tolerance test proved the circular format of circLDLR. ACTB was used as the negative control. (**C**) Three binding sites of miR-1294 on circLDLR predicted by miRanda software. (**D**) The binding site of miR-1294 on CYP19A1 predicted by miRanda software. (**E**) The relative luciferase activity was measured following co-transfection of miR-1294 mimics with the constructs encoding the wild-type or mutant circLDLR cDNA into the KGN cells. (**F**) The relative luciferase activity was measured following co-transfection of miR-1294 mimics with the constructs encoding the wild-type or mutant CYP19A1 3’-UTR into the KGN cells. The firefly luciferase activities were normalized by renilla luciferase activities. The experiments were performed independently in triplicates. * indicates p < 0.05, ** indicates p < 0.01, NS indicates no significant difference. The results are presented as means ±SEM.

To prove the processing characteristics of circLDLR, we further confirmed the sequence of circLDLR by Sanger sequencing (Supplementary Table 7). The results showed that the circLDLR of 295 nt length is formed by backsplicing junction of the 13 and 14 exons of LDLR (Figure 7A). We also performed the RNase R tolerance test by qRT-PCR to prove the circular format of circLDLR (Figure 7B).

According to the prediction, circLDLR has three putative miR-1294 binding sites (Figure 7C). We recombined the circLDLR cDNA (circLDLR-WT), or the mutated cDNA (circLDLR-MUT) losing all the presumed miR-1294 binding sites, to the downstream of the luciferase reporter gene. The vectors were transfected in KGN cells along with the corresponding miRNA mimics. Compared to negative control mimics, miR-1294 mimics significantly reduced luciferase activity with circLDLR-WT by 53%, but not the one with circLDLR-MUT (Figure 7E). This result indicates that miR-1294 directly binds to circLDLR.

In addition, CYP19A1 was a putative target of miR-1294 (Figure 7D). To determine whether CYP19A1 is a direct target of miR-1294, we also constructed luciferase reporter vectors by cloning WT or mutant (loss of miR-1294 binding site) 3’UTR of CYP19A1 downstream of the Renilla luciferase gene and transfected them with miR-1294 mimics into the KGN cells. As shown in Figure 7F, miR-1294 mimics significantly decreased the relative luciferase activity in KGN cells co-transfected with the CYP19A1 WT constructs. The miR-1294 binding site in 3’-UTR is necessary for such regulation, as shown by the CYP19A1 mutant constructs (Figure 7F). Thus, miR-1294 suppressed the expression of CYP19A1 by directly binding to its 3’ UTR.

### Exo- circLDLR inhibited E2 production in KGN cells by regulating miR-1294 and CYP19A1

**Figure 8:**
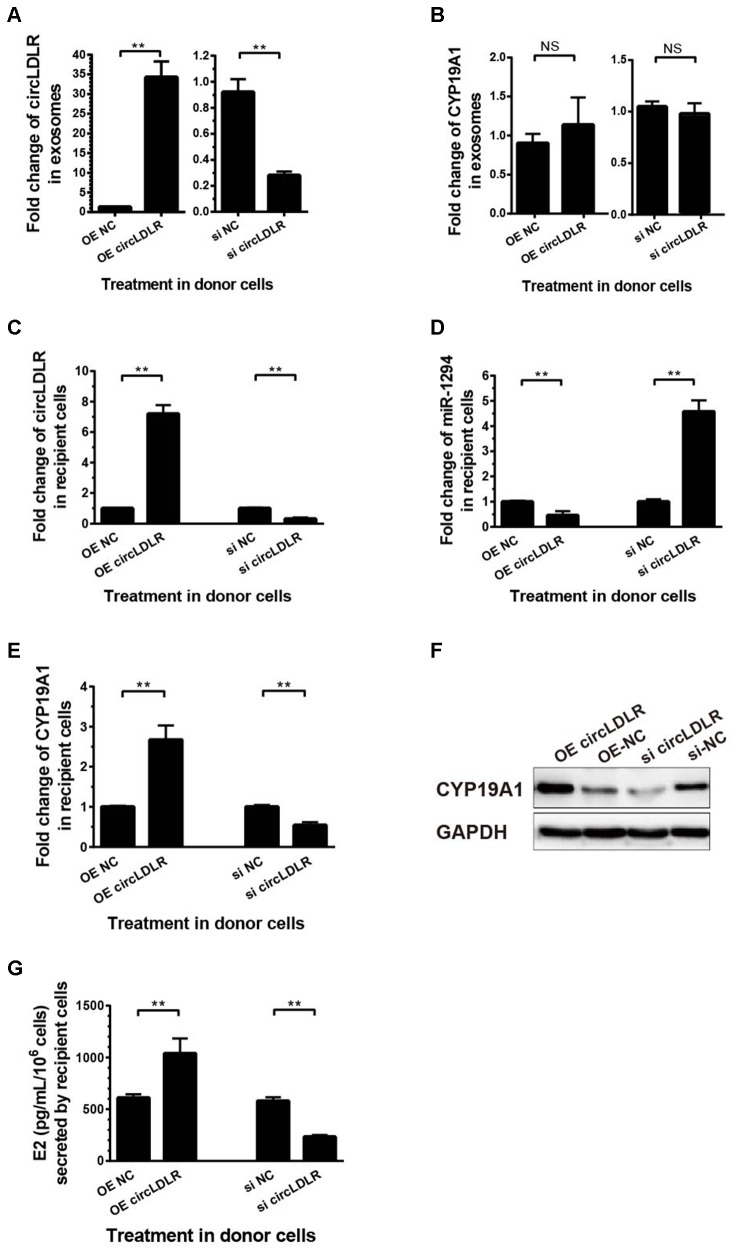
**The regulatory role of circLDLR-miR-1294-CYP19A1 ceRNA network in KGN cells.** (**A**) Overexpression (OE) or siRNA knockdown (si) of circLDLR in KGN donor cells changed circLDLR level in exosomes. (**B**) The abundance of CYP19A1 in exosomes from KGN donor cells with overexpression (OE) or siRNA knockdown (si) of circLDLR. (**C**) The expression level of circLDLR in recipient cells was changed by exosomes derived from donor cells with altered circLDLR expression. (**D**) The expression level of miR-1294 in recipient cells was inversely changed by exosomes derived from donor cells with altered circLDLR expression. (**E**, **F**) CYP19A1 expression in recipient cells was accordingly affected by exosomes derived from donor cells with altered circLDLR expresson at both RNA level (**E**) and protein level (**F**). GAPDH was used as the internal controls for circLDLR and CYP19A1. U6 was used as the internal controls for miR-1294. (**G**) Effects of exosomal circLDLR on E2 production in the recipient KGN cells. Each experiment was performed six times. OE, over expression circLDLR; si, knockdown circLDLR by siRNA; NC, negative control (empty vector or negative siRNA, accordingly). * indicates p < 0.05, ** indicates p < 0.01, NS, no significant difference. The results are presented as means ±SEM.

To determine the regulation of exosomal circLDLR on cellular miR-1294 and CYP19A1, we first modeled the exosomes with altered circLDLR abundance. We transfected the KGN cells with OE- circLDLR vector or si-circLDLR, and quantified the circLDLR and CYP19A1 levels in their secreted exosomes. The abundance of circLDLR in exosome was increased substantially when circLDLR was over-expressed in the donor cells, while was significantly decreased when knocked down in donor cells (Figure 8A). But the abundance of CYP19A1 in exosomes showed no significant changes in both cases (Figure 8B). Next, we examined the downstream effects of these modeled exosomes on the recipient KGN cells. The results showed that exosomes with higher circLDLR abundance dramatically increased (7.2- fold) the circLDLR levels, and the exosomes with depleted circLDLR significantly decreased (3.23- fold) circLDLR level in recipient KGN cells (Figure 8C). Increased circLDLR in exosomes also reduced approximate 2.2- fold of miR-1294 expression in recipient KGN cells, and depleting circLDLR in exosomes increased its level by 4.57-fold (Figure 8D). Moreover, up-regulation or down-regulation of circLDLR in exosomes accordantly altered expression of CYP19A1 in the recipient KGN cells significantly, at both RNA (Figure 8E) and protein levels (Figure 8F). In addition, based on the important role of aromatase in converting androgens to 17-β estradiol (E2), we also measured the E2 production in the medium of the recipient KGN cells treated with exosomes with altered circLDLR abundance. Treated with OE-circLDLR-exosomes, E2 secretion from the recipient KGN cells was significantly elevated (about 2-fold). Meanwhile, the E2 secretion was decreased (2.48- fold) in the medium from the recipient KGN cells treated with si-circLDLR-exosomes (Figure 8G).

**Figure 9:**
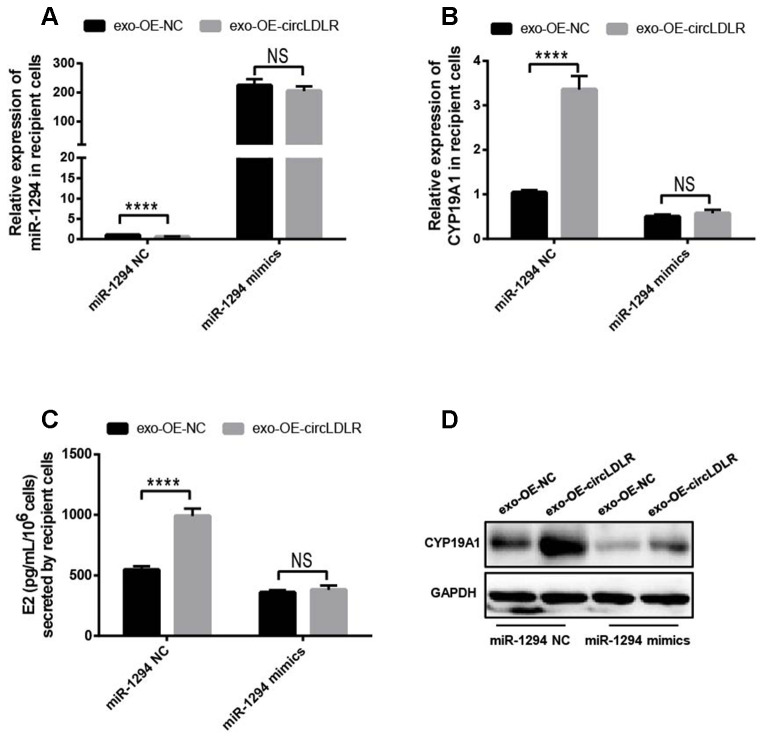
**Confirmation of the direct role of circLDLR in miR-1294/ CYP19A1 axis.** The miR-1294 mimics was transfected into the recipient KGN cells which has been treated with OE- circLDLR exosomes. 48- h later, the expressions of miR-1294 (**A**), CYP19A1 mRNA (**B**) and CYP19A1 protein (**D**) in the corresponding recipient cells were quantified by qRT-PCR and Western blot. At the same time, the estradiol secretions by the recipient cells were also measured by ELISA assay (**C**). GAPDH was used as the internal controls for circLDLR and CYP19A1. U6 was used as the internal controls for miR-1294. Each experiment was performed six times. exo-OE-circLDLR, exosomes with over-expressed circLDLR; exo-OE-NC, negative control exosomes. **** indicates p < 0.0001, ** indicates p < 0.01, NS, no significant difference. The results are presented as means ±SEM.

Moreover, we also performed the withdrawal experiments to prove the exosomal circLDLR functionalities on CYP19A1 expression and estradiol production. As shown in Figure 9A, the decreased expression of miR-1294 in recipient cells treated with OE-circLDLR-exosomes was rescued by miR-1294 mimics co-transfection. And the mRNA and protein levels of CYP19A1 in recipient cells showed no significant difference between the treatments of OE-NC-exosomes and OE-circLDLR-exosomes when miR-1294 mimics was co-transfected (Figure 9B and 9D). At the same time, the estradiol production in the corresponding recipient cell cultures also showed no significant difference (Figure 9C). Therefore, the miR-1294 mimics could rescue the effects of exosomal circLDLR on CYP19A1 expression and activity.

**Figure 10:**
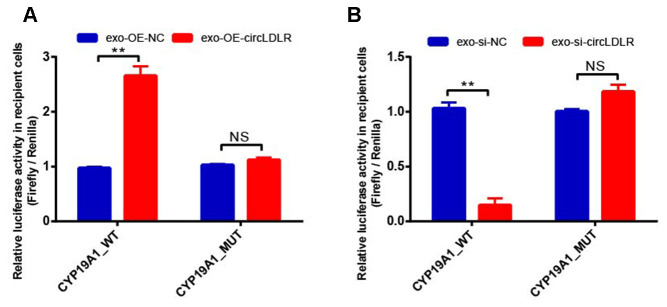
**Validation of exosomal circLDLR regulation on CYP19A1 via miR-1294 by luciferase activity assay.** The relative luciferase activity was measured following transfection of the wild-type or mutant CYP19A1 3’-UTR in recipient KGN cells after treated with (**A**) over-expressed circLDLR exosome (exo-OE-circLDLR) or (**B**) knocked down circLDLR exosome (exo-si-circLDLR), to confirm that exosomal transfer of circLDLR is truly regulatory on CYP19A1 expression via its miR-1294 binding site. The firefly luciferase activities were normalized by renilla luciferase activities. The experiments were performed independently in triplicates. ** indicates p < 0.01, NS indicates no significant difference. The results are presented as means ±SEM.

In addition, OE-circLDLR-exosomes significantly increased (approximate 3- fold) the relative luciferase activity in recipient cells transfected with the CYP19A1 WT constructs but not in the ones transfected with mutant constructs (Figure 10A). On the other hand, si-circLDLR-exosomes significantly decreased the relative luciferase activity in recipient cells transfected with CYP19A1 WT constructs, while no change was observed when they were transfected with the mutant constructs (Figure 10B). The results indicated that exosomal transfer of circLDLR is truly regulatory on CYP19A1 expression via its miR-1294 binding site.

## DISCUSSION

Folliculogenesis is a complex process that involves endocrine and intro-ovarian paracrine interactions to provide the most appropriate microenvironment for oocyte development [40]. Follicular fluid (FF), accumulated inside the antral follicle, represents a very important microenvironment for the development of the oocyte and its biochemical composition reflects the physiological status of the follicle [41]. It is noteworthy that microenvironment signals in follicles are provided not only by gap junctions, paracrine, and autocrine or endocrine signaling factors, but also by exosomes, which constitute a new communication mechanism [42]. In fact, equine, bovine and human studies have all demonstrated the presence of exosomes in FF and proven that exosomes transport RNAs, miRNAs and proteins to recipient cells within the ovarian follicle [18, 20, 43]. The isolation and characterization of these important regulatory molecules from FF exosomes could allow us to clarify unexplored aspects of ovarian physiology [44]. However, the role of FF exosomes in PCOS pathological follicle development has not been clarified yet.

Although various noncoding RNAs are abundant in exosomes [45], previous studies of FF exosomes only focused on exosomal miRNAs [18, 20, 43]. Their results indicated that most of the exosomal miRNAs were predicted to target key elements in pathways related to follicular growth and oocyte maturation in mammals, such as wingless signaling pathway (WNT), transforming growth factor beta (TGF-β), mitogen-activated protein kinase (MAPK), neurotrophin, epidermal growth factor receptor (ErbB) pathways and ubiquitin-mediated pathways. Although circRNA has been recognized as miRNA sponge in various types of cells that leads to the suppression of miRNA function, the exosomal circRNAs have not been examined in follicular fluid until recently [15]. And here, to elucidate the mechanism of the abnormal folliculogensis in PCOS, we reported the circRNA expression profiles in FF exosomes of PCOS patients. A total of 16 differentially expressed circRNAs (5 upregulated and 11 downregulated ones) were identified. According to the GO and pathway analysis of the parental genes of all differentially expressed circRNAs, we found that the most significantly altered pathways were ovarian steroidogenesis, aldosterone synthesis and secretion, thyroid hormone signaling pathway, ubiquitin mediated proteolysis, Jak-STAT signaling pathway, and endocytosis. The results are consistent with the fact that PCOS is accompanied by repression of gene signatures associated with biosynthesis and metabolism of steroids, cholesterol and lipids [46]. Besides ovary-involved pathway, Hepatitis C pathway also showed significant difference between PCOS and control patients. It has been reported that Helicobacter pylori infection is linked to insulin resistance, which is a central factor in the development of PCOS [47]. And, the chlamydial infection-induced chronic inflammation could contribute to both metabolic and hormonal disorders in PCOS patients [48]. A recent publication reported that rats with induced PCOS had different gut microbiota compositions from those of healthy rat. Moreover, improved estrous cycles and reduced androgen production could restore the normal gut microbiota in those PCOS rat. [49]. These findings highlight the need to further study the role of chronic bacterial or virus infection during the development and progression of PCOS.

To identify the role of exosomal circRNAs in PCOS progression, we focused on the circLDLR, which was down-regulated in FF- exosomes of PCOS patients. The results strongly indicated that circLDLR mediated by exosomes inside the ovarian follicle would play an important role in controlling the follicular microenvironment to impact the follicle development in PCOS.

CircRNAs participate in biological processes via multiple mechanisms, including binding RBPs, sponging miRNA, and encoding peptides [50–53]. Thereinto, promoting miRNA degradation through binding to miRNAs is the widely studied biofunction of circRNA [53]. In this study, miR-1294 was predicted as a potential target of circLDLR by bioinformatics analysis, and further luciferase reporter assays verified the direct binding between circLDLR and miR-1294 in KGN cells. Although the role of miR-1294 in follicle development was unclear, previous study had reported that miR-1294 could be the target of circRNAs (i.e. hsa_circ_0004370, hsa_circ_0005198, hsa_circ_0030235) and regulate cell proliferation, apoptosis, migration, and invasion in several kinds of malignancies [26, 54, 55]. Here, we provided novel information that circLDLR directly down-regulated miR-1294 in PCOS follicle development.

Due to the fact that dysregulation of protein expression via different mechanisms plays a key role in the various biological processes, the downstream protein regulated by miR-1294 was further explored. CYP19A1 was predicted as a potential target of circLDLR/miR-1294 pathway, which was also verified by luciferase reporter assay in KGN cells. CYP19A1 (aromatase coding gene) is regarded as an important marker in the etiology of PCOS. Several studies have reported that aromatase gene expression and consequent estradiol production are reduced in preovulatory PCOS follicles compared to healthy controls [56, 57]. The low levels of CYP19A1 mRNA cause the reduction in ovarian aromatase activity and E2 production in various sizes of PCOS follicles [38, 39]. In this situation, the follicles in polycystic ovary arrest at the small antral stage just before the expression of aromatase, thereafter androgen excesses and may contribute to abnormal folliculogenesis [57]. In our study, we also demonstrated that CYP19A1 expression, both in mRNA and protein levels, was significantly lower in granulosa cells of PCOS comparing to the control groups. Moreover, we found a significant positive correlation between the expression of circLDLR and CYP19A. Furthermore, the critical withdrawal experiments and luciferase assay in KGN cells proved that CYP19A1 was involved in circLDLR/miR-1294 pathway as a crucial enzyme through inhibiting E2 production.

In summary, the altered exosomal circRNAs profiles in PCOS follicular fluid were presented, and down-regulated circLDLR was proposed to involve in abnormal ovarian steroidogenesis. We further demonstrated that the circLDLR in exosomes functioned as a vital mediator to regulate E2 secretion via sponging miR-1294 to repress CYP19A1. Our findings broaden the understanding of molecular events in PCOS, and would provide new targets and strategies for further therapies against PCOS.

## MATERIALS AND METHODS

### Participants and sample collection

This study was approved by the Scientific and Ethical Committee of the Shanghai First Maternity and Infant Hospital affiliated to Tongji University. All the participants included in the study were women undergoing IVF or ICSI at Shanghai First Maternity and Infant Hospital between October 2017 and December 2018. A total of 60 participants (30 PCOS and 30 controls) were included in this study after obtaining written informed consent. PCOS patients were diagnosed according to the revised Rotterdam European Society of Human Reproduction and Embryology / American Society for Reproductive Medicine Criteria (2004). The PCOS patients who fulfilled at least two of the following criteria were included: chronic oligo-ovulation or anovulation, androgen excess and polycystic ovaries. The control group contained patients undergoing IVF or ICSI due to male factors or tubal factors of infertility, who had regular menstrual cycles, normal ovary sonographs and normal ovulating. All of the patients had no history of drugs affecting glucose and lipid metabolism and were without any known medical conditions or diseases, including Cushing’s syndrome, congenital adrenal hyperplasia, androgen-secreting tumors and endometriosis. The clinical characteristics of the PCOS and control patients are summarized in Table 2.

**Table 2:** Clinical characteristics of patients.

	**Normal (N=30)**	**PCOS (N=30)**	**P-value**
Age (years)	30.60 ± 1.21	29.6 ± 1.15	NS
BMI (kg/m^2^)	21.31± 0.81	22.06 ± 0.79	NS
FSH (mIU/ml)	6.53±0.72	5.21± 0.92	<0.05
LH (mIU/ml)	6.30 ± 1.14	11.91± 2.16	<0.001
Basal LH/FSH	0.95 ± 0.02	2.23 ± 0.07	<0.001
E2 (pg/ml)	44.5 ± 4.38	69.82 ± 9.8	NS
Testosterone (ng/ml)	0.33 ± 0.05	0.73 ± 0.05	<0.001
Progesterone (ng/ml)	0.58 ± 0.26	0.71 ± 0.13	NS
Antral follicle count	11.2±1.32	23.1±2.71	<0.001
Total FSH administration (IU)	1667±245.1	1589±164.2	NS
Oocytes obtained	8.6 ± 3.01	17.8±6.24	<0.001

Data are the mean ± SEM, NS, not statistically significant.

BMI, body mass index; E2, oestradiol; FSH, follicle-stimulating hormone;

LH, luteotropic hormone; PCOS, polycystic ovary syndrome

All patients underwent controlled ovarian hyperstimulation using a combination of GnRH agonist and recombinant FSH (150–187.5 IU; Gonal-f, Follitropin Alfa, Serono). When two or more follicles were at least 18 mm in diameter and the serum E2 levels were at least 300 pg/mL per dominant follicle, the patients received 250μg hCG (Profasi; Serono). Follicular fluid (2-3 mL) was collected from the dominant follicles (>18 mm) by vaginal puncture under ultrasound echo-guidance 36 h after hCG administration. The samples were centrifuged at 3,000 g for 15 minutes to remove cells debris and other particles. Supernatants were stored at -80°C for further use. At the same time, the granulosa cells (GCs) were collected and then stored at -80°C in the TRIzol reagent (Thermo-Fisher Scientific, USA) to be used for RNA isolation.

### Cell culture

KGN (RCB1154; RIKEN, Wako, Japan) is a steroidogenic human ovarian granulosa tumor cell line. The cells were stored in serum-free cell freezing medium (HAKATA, Chuan Qiu Biotechnology). The cells were cultured in DMEM/F12 medium (Sigma-Aldrich) supplemented with 10% fetal bovine serum and 1% penicillin/streptomycin at 37°C, 5% CO2.

### Exosome isolation

Exosomes from follicular fluid were isolated by ExoQuick-TC Exosome Precipitation Solution Kit (System Biosciences). In brief, 250μL ExoQuick Solution was added to the 1 mL follicular fluid (1:4), mixed well and incubated overnight at 4°C. Then, the mixture was centrifuged at 1,500 g for 30 minutes at 4°C, and the supernatant was removed. The exosome pellet was resuspended in 500μL PBS and passed through a 0.22- μm filter.

### Transmission electron microscopy

Exosomes were analyzed by transmission electron microscopy (TEM) as previously described [58]. A total of 20μL of exosome suspension (5μg/μL) was fixed on a continuous grid and then negatively stained with 2% uranyl acetate solution for 1 minute and air-dried. The samples were observed by FEI Tecnai G1 spirit transmission electron microscope (FEI) at an acceleration voltage of 120 kV.

### Nanoparticle tracking analysis

The numbers and sizes of exosomes were directly measured using the NanoSight NS 300 system (NanoSight Technology, Malvern, UK) [58]. Nanoparticle tracking analysis (NTA) measurements were performed with a 488-nm laser and sCMOS camera module (Malvern Panalytical). Measurements in flow mode were performed with a flow rate of 50, with the flow measurements consisted of 3 measurements of 60 seconds, and the captured data were analyzed using NTA 3.2 software.

### RNA isolation, library construction, and sequencing

Total RNA was extracted from the exosome using TRIzol reagent (Life Technologies, Carlsbad, CA, USA). The concentration of each RNA sample was determined by NanoDrop ND-1000 analysis (Agilent, Wilmington, DE, USA). All RNA samples used in this study passed the quality control based on a qualified ratio of OD_260_ to OD_280_ (1.8-2.1).

RNA library preparation and circRNA sequencing were performed by CloudSeq Biotech (Shanghai, China). The rRNA was depleted from total RNA using the Ribo-Zero Magnetic Gold Kit (Epicenter, Madison, WI, USA). The rRNA-depleted RNA was used to construct the RNA libraries with TruSeq Stranded RNA Library Prep Kit (Illumina, San Diego, CA, USA). Briefly, rRNA-depleted RNA was used for first- and second- strand cDNA synthesis with random hexamer primers. For second- strand cDNA synthesis, dUTP mix (without dTTP) was used which allows for the removal of the second strand. The cDNA fragments were then end-repaired, dA-tailed and adapter ligated. Ligated cDNA products were purified and treated with uracil DNA glycosylase to remove the second-strand cDNA. Purified first-strand cDNA was subjected to 14 cycles of PCR amplification. The library quality was evaluated with BioAnalyzer 2100 system (Agilent Technologies, Richardson, TX, USA). The strand-specific RNA-seq libraries were sequenced on Illumina HiSeq sequencer in 150 bp paired end mode. Similar sequencing depths (from 60M to 82M reads, averaged at 70M reads) were achieved from different RNA libraries. Reads were mapped to human reference genome GRCh37/hg19 with STAR software, and the output file of STAR was analyzed with DCC software to identify circRNAs. Raw junction reads for all samples were normalized by total mapped reads count and log2 transformed. Differentially expressed circRNAs were identified by edgeR package (http://www.rproject.org/) between two groups. A circRNA candidate was considered to be differentially expressed if its levels between two experiment groups showed a statistically significant difference (p<0.05) of at least two-fold. The FDR correction for p values and PCR validations were used to confirm the high-throughput analysis. To generate the overview of differentially expressed circRNAs between exo-PCOS and exo-control, the hierarchical clustering analysis was performed based on the expression levels of all identified circRNAs and the significant difference between exo-PCOS and exo-control by Cluster and TreeView software.

### Gene ontology (GO) and KEGG pathway analyses

GO and KEGG analyses were performed for the differentially expressed circRNA-associated genes. GO analysis was performed to describe genes and gene product attributes in any organism (http://www.geneontology.org). This ontology covers three domains: biological processes, cellular components and molecular functions. The p value denotes the significance of the GO term enrichment among differentially expressed genes (p value ≤ 0.05 is considered as significant). For pathway analysis, we used the web-based Molecular Annotation System 3.0 (MAS 3.0; http://bioinfo.capitalbio.com/mas3/), which integrated three different open-source pathway resources: KEGG, BioCarta and GenMAPP. The significantly altered pathways were selected using the threshold of the p value and FDR (corrected p value) < 0.05 derived from the hypergenomic test.

### Construction of the circLDLR-mediated ceRNA network based on sequencing data

A potential circLDLR-mediated ceRNA network was constructed based on sequencing data to investigate whether circLDLR functions as a miRNA sponge. The putative miRNAs and mRNAs included in the construction of the circLDLR-mediated ceRNA network are as follows: (i) miRNA selection: miRNAs (top 5-6) were predicted to possess circLDLR binding sites using miRanda v3.3a software. (ii) mRNA selection: the target genes of the candidate miRNAs were predicted using miRbase (http://www.mirbase.org/) and compared with the genes involved in the ovarian steroidogenesis pathway. The selected mRNAs met two criteria: 1) putative target genes of miRNAs with MREs in circLDLR; and 2) the genes were putative to involve in ovarian steroidogenesis pathway. The network of circRNAs and their downstream miRNAs was generated by cytoscape software (v2.8.0).

### qRT-PCR

Total RNA was extracted from the exosomes, granulosa cells, or cultured KGN cells using TRIzol Reagent (Invitrogen) according to the manufacturer’s protocol. The SuperScriptTM III Reverse Transcriptase (Invitrogen) and qPCR SYBR Green master mix (CloudSeq) were used for mRNA and circRNA qRT-PCR. The Hairpin-it^TM^ microRNA and U6 snRNA Normalization RT-PCR Quantitation Kit (GenePharma) was used for miRNA qRT-PCR. qRT-PCR were performed on an Applied Biosystems AB7500 Real-Time PCR system. Each set of qRT-PCRs was repeated at least three times, and the fold change in the expression of each gene was analyzed using the ΔΔCt method [59]. U6 snRNA was used as an internal control for miRNAs, and the circRNA and mRNA levels were normalized to GAPDH. qRT-PCR primers of circRNAs, miRNAs and mRNAs are listed in Supplementary Table 1.

### Western blot analysis

Expression of CD63, CD81, Calnexin and CYP19A1, was assessed by Western blot analysis, and the samples were normalized to GAPDH. Total protein was prepared using RIPA buffer. The protein lysate was separated by 12% SDS-PAGE and transferred onto PVDF membranes (EMD Millipore, Billerica, MA, USA). Following blocking for 1 h, the membranes were incubated with primary antibodies, such as anti-CD63 (ab216130, 1:1000, Abcam,), anti-CD81 (ab109201, 1:1000, Abcam,), anti-Calnexin (ab125011, 1:1000, Abcam), anti-CYP19A1 (#14528, 1:1000, CST), and anti-GAPDH (#5174, 1:4000; CST), overnight at 4 °C and then incubated with the corresponding secondary antibodies at 1:2000 dilution for 2 h at room temperature.

### Luciferase reporter constructs and luciferase activity assay

The direct interaction between the partners of circLDLR-mediated ceRNA network was evaluated by luciferase activity assay. A luciferase reporter vector (GP-mirGLO Dual-Luciferase miRNA Target Expression Vector; Promega) was used for luciferase constructs. circLDLR and the 3’ UTR of CYP19A1 were cloned by RT-PCR. CircLDLR-WT, circLDLR-mutant and CYP19A1-3’UTR (WT and mutant) were constructed as our previously reported [60, 61]. KGN cells were seeded onto 24-well plates and allowed to grow for ~24 h without antibiotics before transfection. The constructed reporter vectors (300 ng) were transfected into cells together with the miRNA (miR-1294) mimics or negative control mimics (100 nM) using Lipofectamine 2000 (2μL). Cells were lysed ~24 h after transfection, and the luciferase activity was assayed using the Dual-Luciferase Reporter Assay System (Promega). Firefly luciferase activities were normalized to Renilla luciferase activities. In addition, the relative luciferase activity was measured following co-transfection of exosomal circLDLR (over-expressed circLDLR or knocked down circLDLR) with the wild-type or mutant CYP19A1 3′-UTR in recipient KGN cells to confirm that exosomal transfer of circLDLR is truly functional and essential to CYP19A1 down- or up-regulation rather than other confounding factors. All the experiments were performed independently in triplicates.

### Preparation of exosomes with over-expressed circLDLR (OE- circLDLR) or knocked down circLDLR (si-circLDLR)

The circLDLR overexpressing plasmid (OE- circLDLR) and circLDLR siRNA (si- circLDLR) were constructed by Gene Pharma (Shanghai, China). For OE- circLDLR, the GV485- circLDLR vector was constructed by inserting circLDLR PCR amplicon into KpnI/BamHI-digested GV485. To knock down the expression of circLDLR, circLDLR siRNA was synthesized with the sequence as follows: 5’-UGCCUCACAGGACAAAGUA-3’. KGN cells were transfected with OE- circLDLR or si- circLDLR 24h after cultured without antibiotics, and exosome were isolated from medium 48h later. To determine the effect of overexpression or knock-down in exosomes, the expression levels of circLDLR and CYP19A1 in exosomes of KGN cells which transfected with OE- circLDLR or si- circLDLR were quantified by qRT-PCR.

### Cell transfection

The exosomes derived from the KGN cells transfected with OE-circLDLR or si-circLDLR were collected. To assess the effects of exosomal circLDLR on CYP19A1 expression and estradiol secretion, about 5×10^4^ KGN cells/well were seeded in a 6- well plate with complete DMEM/F12. After 24-h, the cells were starved for 12- h and then treated with the exosomes (100 μg/mL) of OE-circLDLR or si-circLDLR. After 48- h, the expressions of miR-1294, CYP19A1(mRNA and protein levels) in the corresponding treated KGN cells were quantified by qRT-PCR and Western blot analysis. At the same time, the estradiol secretion in the corresponding treated KGN cells cultures were also quantified by ELISA assay. Each experiment was performed six times.

In addition, to further prove whether the role of exosomal circLDLR in regulating CYP19A1 expression could be rescued by the putative miRNA (miR-1294), the miR-1294 mimics (forward: 5’-UGUGAGGUUGGCAUUGUUGUCU-3’; reverse: 5’-ACAACAAUGCCAACCUCACA-3’, Gene Pharma, Shanghai, China) was transfected with the treatment of OE-circLDLR exosomes into the recipient KGN cells. A pre-designed negative control (forward: 5’-UUCUCCGAACGUGUCACGUTT-3’; reverse: 5’-ACGUGACACGUUCGGAGAATT-3’) was used as the negative controls. After transfection, the expression of miR-1294, CYP19A1 and the estradiol secretion in the recipient cells were quantified.

### Assay for E2 secretion

Estradiol secretion of the KGN cells were determined by testing E2 accumulation in the culture medium. In detail, the KGN cells were plated onto 24-well plates at a density of 1×10^6^ viable cells per well and were incubated with 10^−5^ M of T (testosterone, substrate for E2) and hMG (500 mIU/mL). Next, the media were collected after 48 h, and the E2 concentrations were measured by means of the enzyme immunoassay (EIA) Kit (Amerlite P, Amersham Pharmacia, Tokyo, Japan). The intra-assay and inter-assay coefficients of variation were <10%. Each experiment was performed six times independently.

### Statistical analysis

All statistical analyses were performed using SPSS 17.0 software (SPSS Inc.) unless otherwise noted. Differences in the expression levels of the partners of the circLDLR-mediated ceRNA network in FF exosome or granulosa cell samples were evaluated by two-tailed t-test. Differences were considered statistically significant at *p* < 0.05.

## Supplementary Material

Supplementary Tables

Supplementary Table 2

Supplementary Table 3

Supplementary Table 4

Supplementary Table 5

Supplementary Table 6
