# Supplementary material for: Depletion of exosomal circLDLR in follicle fluid derepresses miR-1294 function and inhibits estradiol production via CYP19A1 in polycystic ovary syndrome
Source: Aging (Albany NY). 2020 Jul 10;12(15):15414–35. doi: 10.18632/aging.103602 (PMC7467373; doi:10.18632/aging.103602)
Supplement: Supplementary Tables [file aging-12-103602-s006..pdf]

SUPPLEMENTARY TABLES

Supplementary Table 1. Sequences of PCR primers.

Gene	Forward primer (5'-3')	Reverse primer (5'-3')
chr19:11230768-11231198 (hsa_circ_0006877/circLDLR)	GCGGCTGCCAGTATCTGT	TTGGCTGGGTGAGGTTGT
chrM:2262-2426 (novel)	AGGCTTATGCGGAGGAGAA	GCGTCAGATCAAAACACTGAA
chr13:110076504-110076682 (novel)	AGGGCTAAGCATAGTGGGG	ACCTGGCGGTGCTTCATA
chr15:68352856-68353111 (novel)	TGCCATTTAAATTGGGAGG	GGCACGGTCTCAGCTCTC
chr17:16075117-16097953 (hsa_circ_0005069)	GCTGCGGTTTTGCCTTTA	AATGCTCCTTGGTTGGGA
CYP19A1	GAGAATTCATGCGAGTCTGGA	CATTATGTGGAACATACTTGAGG ACT
miR-1294	TGTGAGGTTGGCATTG	GTCGTATCCAGTGCGTGTCGTGG AGTCGGCAATTGCACTGGATACG ACagacaa
GAPDH	CGCTGAGTACGTCGTGGAGTC	GCTGATGATCTTGAGGCTGTTGTC
ACTB	GGATCAGCAAGCAGGAGT	AAAGCCATGCCAATCTCATC
U6	CTCGCTTCGGCAGCACA	AACGCTTCACGAATTTGCGT

Please browse Full Text version to see the data of Supplementary Tables 2 to 6.

Supplementary Table 2. The GO analysis of the coding genes of up-regulated circRNAs.

Supplementary Table 3. The GO analysis of the coding genes of down-regulated circRNAs.

Supplementary Table 4. KEGG pathway analysis of the coding genes of dysregulated circRNAs.

Supplementary Table 5. The miRNAs predicted to possess MREs in circLDLR.

Supplementary Table 6. The genes associated with hsa_circ_0006877-miRNA-mRNA ceRNA network.

Supplementary Table 7. The Sanger sequence of hsa_circ_0006877 (chr19:11230768-11231198+).

<table>
<tr>
<td>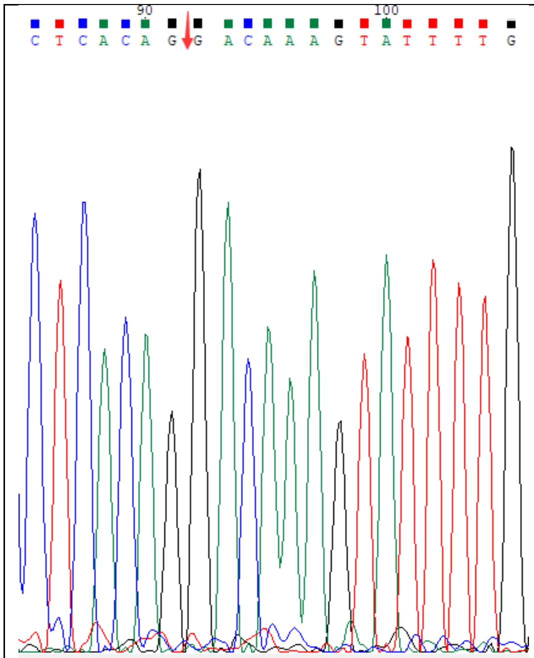
</td>
<td>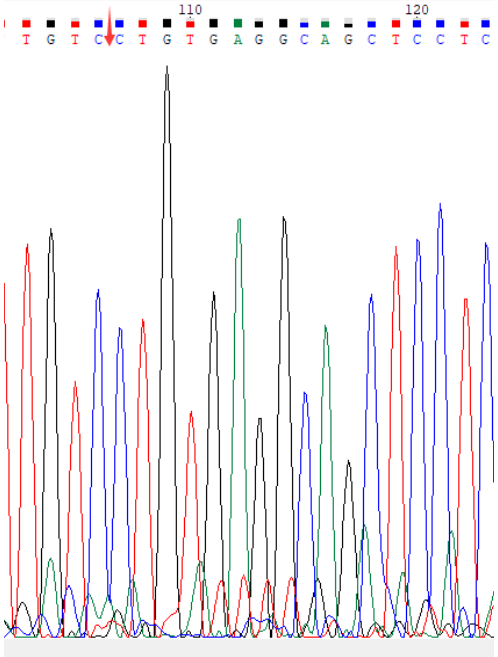
</td>
</tr>
<tr>
<td>>3F_.ab1
GGGGGGTGAGATCACCCCCACTCGCCCAAGTTTACC
TGCGCCTGCCCGGACGGCATGCTGCTGGCCAGGGAC
ATGAGGAGCTGCCTCACAGGACAAAGTATTTTGGAC
AGATATCATCAACGAAGCCATTTTCAGTGCCAACCGC
CTCACAGGTTCCGATGTCAACTTGTTGGCTGAAAACC
TACTGTCCCCAGAGGATATGGTCCTCTTCCACAACCT
CACCCAGCCAAA</td>
<td>>3R_.ab1
CGTTCTCTGCGGCGTAGGTTTTCAGCCACAAGTTGAC
ATCGGAACCTGTGAGGCGGTTGGCACTGAAAATGGC
TTCGTTGATGATATCTGTCCAAAATACTTTGTCCTGT
GAGGCAGCTCCTCATGTCCCTGGCCAGCAGCATGCC
GTCCGGGCAGGCGCAGGTAAACTTGGGCGAGTGGGG
GTTGATCTGCGGGGCAGGGAGGCACAGATACTGGC
AGCCGCAACCTCCCC</td>
</tr>
<tr>
<td colspan="2">>chr19:11230768-11231198+_for PCR
AACTGGTGTGAGAGGACCACCCTGAGCAATGGCGGCTGCCAGTATCTGTGCCTCCCTGCCCCGCAGATCAACCC
CCACTCGCCCAAGTTTACCTGCGCCTGCCCGGACGGCATGCTGCTGGCCAGGGACATGAGGAGCTGCCTCACAG
GACAAAGTATTTTGGACAGATATCATCAACGAAGCCATTTTCAGTGCCAACCGCCTCACAGGTTCCGATGTCAACT
TGTTGGCTGAAAACCTACTGTCCCCAGAGGATATGGTTCTCTTCCACAACCTCACCCAGCCAAGAGGAGTG
*Primer sequences in green; splicing site in red</td>
</tr>
</table>
